# Echocardiographic reference ranges for noninvasive left ventricular 18-segment myocardial work index and work efficiency in a healthy Asian population

**DOI:** 10.1186/s12947-023-00299-4

**Published:** 2023-01-23

**Authors:** Jian Wu, Xinyi Huang, Kunhui Huang, Yiruo Tang, Qiumei Gao, Xu Chen, Bo Jing, Xinyu Wang, Biqin Lin, Maolong Su

**Affiliations:** 1 Department of Ultrasonography, School of Medicine, Xiamen Cardiovascular Hospital of Xiamen University, Xiamen University, Xiamen, China; 2 School of Medicine, Xiamen University, Xiamen, China; 3 Xiamen Key Laboratory of Precision Medicine for Cardiovascular Disease, Xiamen, China; 4 Department of Ultrasonography, Xiamen Humanity Hospital, Fujian Medical University, Xiamen, China

**Keywords:** Myocardial work, Echocardiography, Left ventricular function, Normal population

## Abstract

**Background:**

Left ventricular (LV) myocardial work index (WI) and work efficiency (WE) have become the latest indicators for assessing LV function. Reference ranges for normal LV segmental WI and WE have not been established.

**Methods:**

Four hundred eleven healthy Asian subjects (47% men, median age: 35 years) were enrolled prospectively. WI and WE were analysed using the LV pressure–strain loop (LVPSL) with specific software.

**Results:**

WI and WE differed significantly between segments as well as between walls and levels of the left ventricle. The anteroseptal basal segment had the lowest WI and WE (1440 mmHg ± 324 and 92% [88–96], respectively) among the eighteen segments. Significant WI and WE differences were found between sexes and age groups. No correlation was observed between age groups and the average WI of any wall or level in men, while the average WI of several different walls and levels in women showed significant differences between age groups. The average WI of most walls and levels increased with age in women. No correlation was found between age groups and the average WE of any wall or level in either men or women.

**Conclusions:**

This study establishes the normal reference values of WI and WE of eighteen segments for clinical work and clinical experiments. There were significant differences in WI and WE between segments, levels, and walls of the normal left ventricle. Sex should be considered when analysing WI and WE. Age should be considered when analysing WI in women.

**Graphical Abstract:**

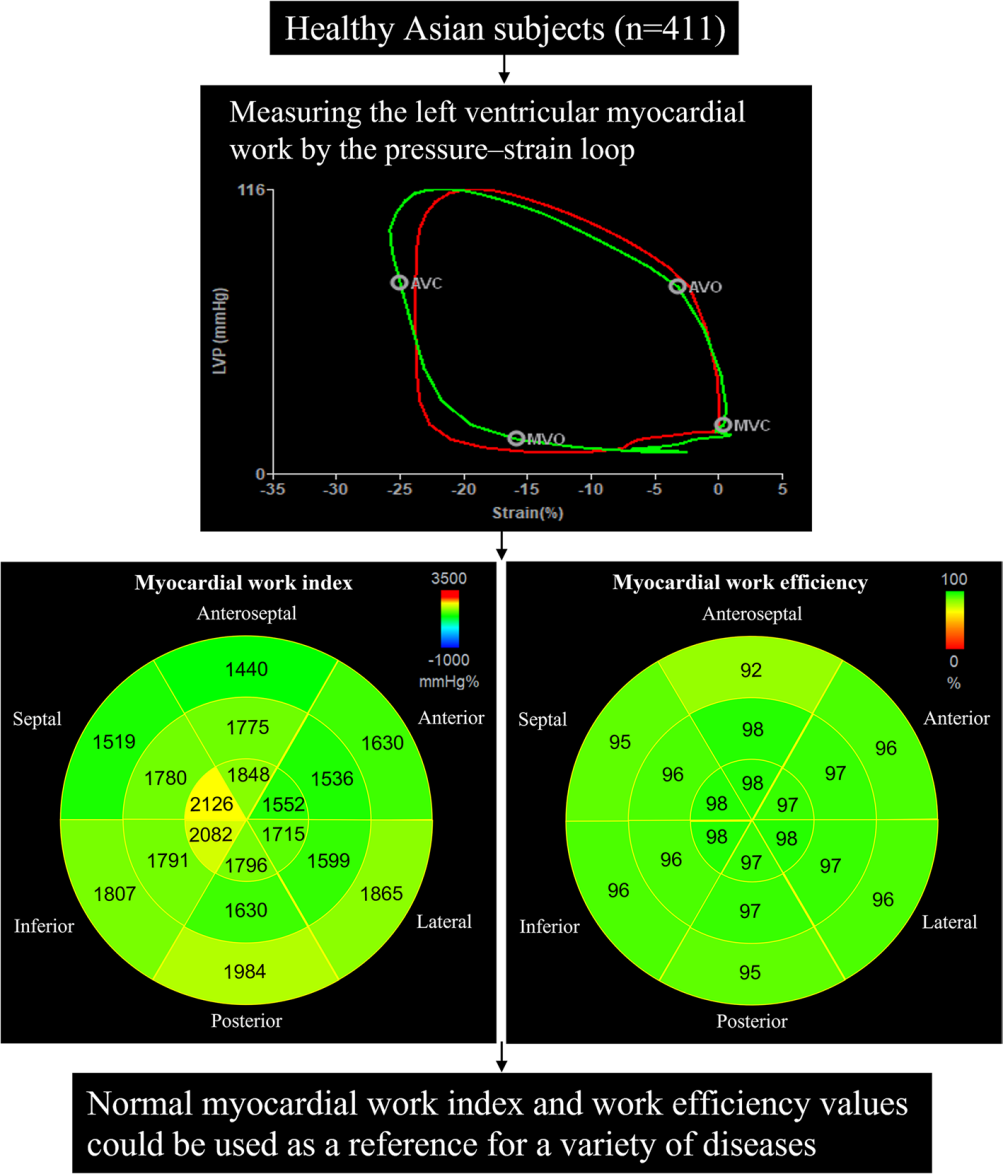

**Supplementary Information:**

The online version contains supplementary material available at 10.1186/s12947-023-00299-4.

## Background

Noninvasive left ventricular (LV) myocardial work (LVMW) is a novel and reliable indicator for assessing LV systolic function and has been used in several experimental and clinical works [1–7]. Noninvasive LVMW is obtained by integrating the LV longitudinal strain (LS), mitral and aortic valvular events, and brachial artery pressures using specific software. Normal reference values of global WI and global WE were analysed previously; however, normal reference values of different segmental WI and WE have not been studied. A previous study confirmed that there are significant differences in the LS of different LV segments in a healthy population [8]. Noninvasive myocardial work is derived based on LS; theoretically, there could be differences in the myocardial work of each segment of the left ventricle in healthy subjects.

The study aimed to 1) establish normal reference values ​​for WI and WE of different segments in a healthy Asian population; 2) explore the differences in WI and WE of different segments; and 3) explore the implications of sex and age on WI and WE.

## Methods

### Population

A total of 452 healthy Asian subjects (age range, 18–65 years) were prospectively recruited from Xiamen Cardiovascular Hospital of Xiamen University between April 2021 and July 2021. The recruited population included hospital staff, people who came to this hospital for medical check-ups and their families, and people who came here for training or visits. The inclusion criteria of this study were as follows: age ≥ 18 years, body mass index < 30 kg/m^2^, normal physical examination results, normal electrocardiogram results, normal two-dimensional echocardiography (2DE) results, and absence of cardiovascular or respiratory diseases. The Institutional Ethics Committee approved the protocol, and all subjects provided informed consent.

### Echocardiographic data acquisition

2DE and four-dimensional echocardiography (4DE) LV images were performed with a Vivid E95 system (GE Vingmed Ultrasound, Horten, Norway) with an M5Sc probe and a 4Vc probe, respectively. All datasets were acquired using electrocardiogram gating over three to five cardiac cycles following the protocols [9, 10]. Data were stored digitally for offline analysis.

### Echocardiographic measurements

Standard measurements were performed using software (EchoPAC V.204, GE) in accordance with the guidelines [10].

Quantitative parameters of the left ventricle and left atrium were analysed using 4DE images by the 4D Auto LVQ software package and 4D Auto LAQ software package, respectively; LV end-diastolic volume, LV end-systolic volume, LV ejection fraction, and maximum and minimum volumes of the left atrium were automatically obtained. The transmitral E- and A-wave velocities were obtained by pulse-wave Doppler from the apical four-chamber view. The early diastolic velocities (e’) were measured by pulse-wave tissue Doppler from the apical four-chamber view. LV LS was acquired using three standard LV apical views with a frame rate ≥ 60 frames/s.

LVMW was measured by an LV pressure–strain loop (LVPSL). The mitral and aortic valve event timings were determined by visualization of the apical three-chamber views. LVPSL was generated by integrating the LV LS, valve event timings, and blood pressure readings using the software. The validation of LVMW was performed in several studies [1, 11, 12].

Four LVMW indices were obtained by LVPSL:

(i) Work index (WI): the LVMW derived from the area of LVPSL.
(ii) Constructive work (CW): positive work during shortening in systole and work during lengthening during isovolumic relaxation (IVR).
(iii) Wasted work (WW): negative work during lengthening in systole and work during shortening during IVR.
(iv) Work efficiency (WE): CW/(CW + WW).

**Fig. 1:**
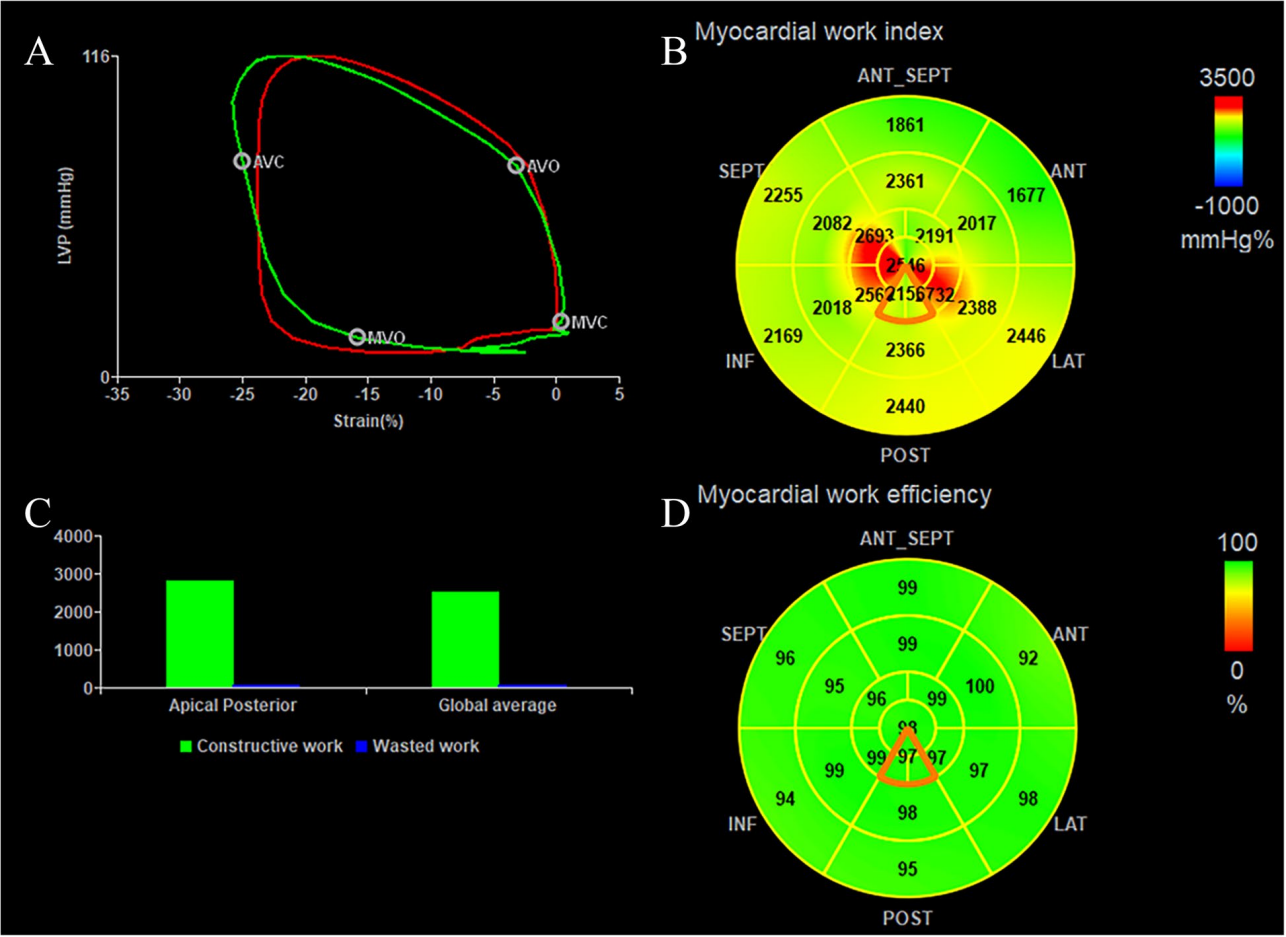
**A** Noninvasive left ventricular pressure–strain loop diagram of a normal subject. The red and green loop areas represent the average global myocardial work index and the represented myocardial work index of the posterior apical segment, respectively. **B** 18-segment bull's-eye expression of myocardial work index. **C** Bar graph representing constructive work and wasted work. **D** 18-segment bull's-eye expression of myocardial work efficiency

WI and WE were calculated for each LV segment in the software (according to the 18-segment model) [13] (Fig. 1).

### Statistical analysis

All statistical analyses were performed using SPSS version 26 (SPSS Inc., IBM Corp). *P* < 0.05 was considered significant. All data normality was tested by the Kolmogorov–Smirnov test. Data are expressed as the mean ± standard deviation (SD) or median (interquartile range) as appropriate. The 95% confidence interval for WI was calculated as the mean ± 1.96 SD. The lowest (2.5th percentile) expected values for WE were computed using a bootstrap of 1000 samples.

Unpaired *t* tests or one-way ANOVA were used to compare normally distributed data. The Mann–Whitney *U* test or Kruskal–Wallis test was used to compare nonnormally distributed data. Correlations between variables were assessed using Spearman correlations.

The intra- and inter-observer variabilities of WI and WE were tested in twenty random individuals using the intraclass correlation coefficients and Bland–Altman analysis. The intra-observer analysis was performed after a 2-week interval. The inter-observer analysis was performed by a second independent blinded observer.

## Results

### Clinical and echocardiographic characteristics

Forty-one individuals were excluded because of poor image qualities of 2DE or 4DE images. Thus, the feasibility of LVMW measurement was 90.9% in this study. Table 1 summarizes the demographic and echocardiographic data of the enrolled population. LV global LS (LV GLS), LV global WI, LV global CW, and LV global WE were higher in women than in men (*P* < 0.001), while LV global WW was lower in women than in men (*P* = 0.005).

**Table 1:** Clinical and echocardiographic characteristics of the study population

Parameters	Total (*n* = 411),	Men (*n* = 195)	Women (*n* = 216)	*P*-value*
Age (years)	35 (29–45)	34 (28–43)	37 (29–46)	0.056
Height (cm)	164 (159–171)	171 (168–176)	159 (156–163)	< 0.001
Weight (kg)	62 (54–70)	69 (64–75)	54 (50–60)	< 0.001
BMI (kg/m^2^)	22.6 (20.6–24.8)	23.5 (22.0–25.4)	21.6 (19.8–23.9)	< 0.001
BSA (m^2^)	1.67 (1.54–1.80)	1.80 (1.74–1.89)	1.56 (1.48–1.63)	< 0.001
SBP (mmHg)	119 (110–128)	123 (116–130)	114 (105–125)	< 0.001
DBP (mmHg)	72 (65–78)	73 (67–78)	71 (63–77)	0.011
Heart rate (bpm)	68 (62–73)	65 (61–73)	69 (63–73)	0.012
LV EDV (ml)	94 (88–105)	104 (97–110)	89 (84–93)	< 0.001
LV ESV (ml)	33 (29–38)	38 (34–42)	30 (27–33)	< 0.001
LV EF (%)	65 (63–68)	64 (61–66)	66 (64–68)	< 0.001
E wave (m/s)	0.78 (0.69–0.92)	0.74 (0.64–0.84)	0.84 (0.74–0.95)	< 0.001
A wave (m/s)	0.55 (0.46–0.65)	0.53 (0.44–0.63)	0.56 (0.47–0.66)	0.013
Transmitral E/A ratio	1.4 (1.2–1.8)	1.4 (1.16–1.73)	1.6 (1.2–1.8)	0.024
Septal e' wave (m/s)	11 (10–13)	11 (10–13)	11 (10–13)	0.997
Lateral e' wave (m/s)	14 (13–17)	14 (12–17)	15 (13–17)	0.388
E/e' ratio	6.1 (5.1–7.2)	5.7 (4.9–6.8)	6.5 (5.6–7.6)	< 0.001
LA max (ml)	40 (36–45)	43 (40–47)	37 (34–41)	< 0.001
LA min (ml)	19 (17–22)	21 (19–23)	18 (16–21)	< 0.001
LV GLS (%)	-19.6 (-21.1– -18.1)	-18.3 (-19.6– -17.4)	-20.7 (-21.8– -19.5)	< 0.001
LV global WI (mmHg%)	1749 ± 231	1676 ± 211	1814 ± 228	< 0.001
LV global CW (mmHg%)	2019 ± 265	1946 ± 224	2085 ± 283	< 0.001
LV global WW (mmHg%)	79 (56–105)	81 (63–108)	74 (52–99)	0.005
LV global WE (%)	96 (94–97)	95 (94–96)	96 (95–97)	< 0.001

Data are displayed as mean ± SD or median (interquartile range), appropriately. *BMI* body mass index, *BSA* body surface area, *CW* constructive work, *DBP* diastolic blood pressure, *EDV* end-diastolic volume, *EF* ejection fraction, *ESV* end-systolic volume, *GLS* global longitudinal strain, *LA* left atrium, *LV* left ventricular, *SBP* systolic blood pressure, *WE* work efficiency, *WI* work index, *WW* wasted work. *P-value refers to sex differences

### Functional nonuniformity

**Fig. 2:**
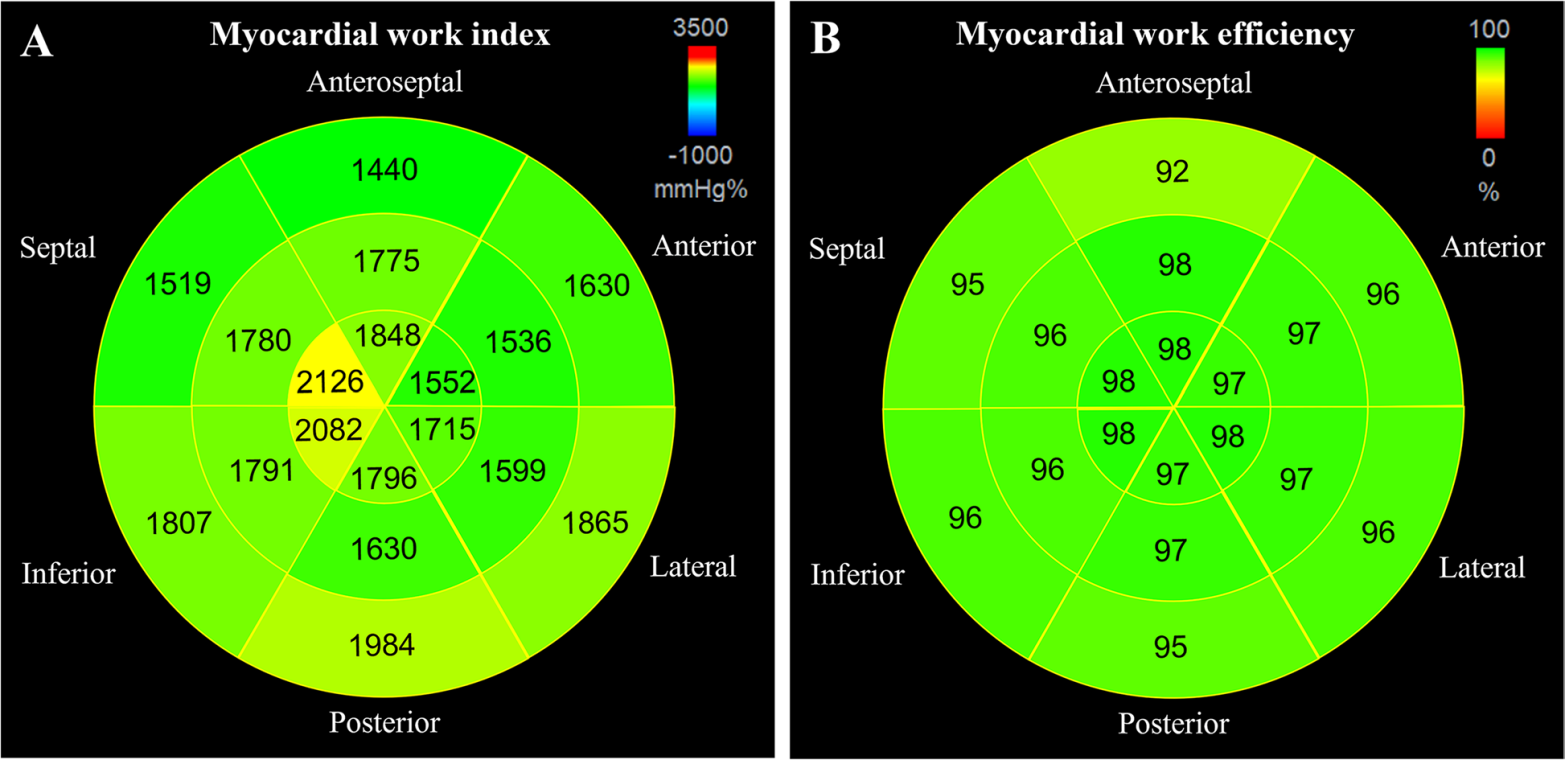
**A** 18-segment bull's-eye diagram shows the functional nonuniformity of the myocardial work index in the normal left ventricle. The values in the different segments are mean values. **B** 18-segment bull's-eye diagram shows the functional nonuniformity of myocardial work efficiency in the normal left ventricle. The values in the different segments are median values

Table 2 and Table 3 summarize the WI and WE of different segments, levels, and walls of the population. Figure 2 displays the mean values of WI and the median values of WE for the 18 segments. Functional nonuniformity was found for all WIs and WEs in the normal left ventricle. WI and WE differed significantly between different segments, as well as different walls and levels of the left ventricle.

**Table 2:** Comparisions of normal values of segmental work index

	All levels	Basal	Middle	Apical	*P*-value (levels)
All walls' WI (mmHg%)	–	1694 (1387–2007)	1689 (1427–1952)	1870 (1531–2179)‡§	< 0.001
Anteroseptal WI (mmHg%)	1688 ± 396	1440 ± 324^a^	1775 ± 343†	1848 ± 391‡§	< 0.001
Septal WI (mmHg%)	1808 ± 432	1519 ± 341	1780 ± 339†	2126 ± 378‡§	< 0.001
Inferior WI (mmHg%)	1894 ± 428	1807 ± 438	1791 ± 371	2082 ± 410‡§	< 0.001
Posterior WI (mmHg%)	1803 ± 409	1984 ± 374	1630 ± 363†	1796 ± 410‡§	< 0.001
Lateral WI (mmHg%)	1726 ± 430	1865 ± 396	1599 ± 401†	1715 ± 449‡§	< 0.001
Anterior WI (mmHg%)	1573 ± 389	1630 ± 399	1536 ± 373†	1552 ± 387‡	0.001
*p*-value (walls)	< 0.001	< 0.001	< 0.001	< 0.001	–

Data are displayed as mean ± SD or median (interquartile range), appropriately. †*P*-value < 0.05 between basal level and middle level. ‡*P*-value < 0.05 between basal level and apical level. §*P*-value < 0.05 between middle level and apical level. ^a^Anteroseptal basal WI was significantly lower than any other segmental WI

**Table 3:** Comparisions of normal values of segmental work efficiency

	All levels	Basal	Middle	Apical	*P*-value (levels)
All walls' WE (%)	–	95 (92–98)	97 (94–99)†	98 (95–99)‡§	< 0.001
Anteroseptal WE (%)	96 (93–99)	92 (88–96)^a^	98 (95–99)†	98 (96–99)‡	< 0.001
Septal WE (%)	97 (94–99)	95 (91–98)	96 (93–98)†	98 (97–99)‡§	< 0.001
Inferior WE (%)	97 (94–98)	96 (94–98)	96 (94–98)	98 (95–99)‡§	< 0.001
Posterior WE (%)	96 (93–98)	95 (92–97)	97 (93–99)†	97 (94–99)‡§	< 0.001
Lateral WE (%)	97 (95–99)	96 (94–98)	97 (95–99)†	98 (95–99)‡	< 0.001
Anterior WE (%)	97 (93–98)	96 (92–98)	97 (94–99)†	97 (94–99)‡	< 0.001
*p*-value (walls)	< 0.001	< 0.001	< 0.001	< 0.001	–

Data are displayed as median (interquartile range). †*P*-value < 0.05 between basal level and middle level. ‡*P*-value < 0.05 between basal level and apical level. §*P*-value < 0.05 between middle level and apical level. ^a^Anteroseptal basal WE was significantly lower than any other segmental WE

### Normal reference values

The values of WI and WE are summarized in Table 4 and Table 5.

**Table 4:** Parameters of left ventricular segmental WI according to sex

	Men, mean ± SD	Men, 95% CI	Women, mean ± SD	Women, 95% CI	*P*-value*
Anteroseptal WI (mmHg%)					
Basal	1378 ± 313	765–1991	1497 ± 325	807–2187	< 0.001
Middle	1716 ± 332	1065–2367	1828 ± 346	1150–2506	0.001
Apical	1857 ± 395	1083–2631	1841 ± 389	1079–2603	0.677
Septal WI (mmHg%)					
Basal	1446 ± 327	805–2087	1586 ± 341	918–2254	< 0.001
Middle	1694 ± 343	1022–2366	1859 ± 316	1240–2478	< 0.001
Apical	2063 ± 359	1359–2767	2182 ± 386	1039–3325	0.001
Inferior WI (mmHg%)					
Basal	1723 ± 424	892–2554	1883 ± 438	1025–2741	< 0.001
Middle	1742 ± 377	1003–2481	1835 ± 360	1129–2541	0.011
Apical	2047 ± 403	1257–2837	2114 ± 414	889–3339	0.100
Posterior WI (mmHg%)					
Basal	1910 ± 384	1157–2663	2050 ± 352	1360–2740	< 0.001
Middle	1510 ± 335	853–2167	1739 ± 354	1045–2433	< 0.001
Apical	1757 ± 385	1002–2512	1830 ± 429	989–2671	0.069
Lateral WI (mmHg%)					
Basal	1738 ± 366	1021–2455	1979 ± 388	1289–2669	< 0.001
Middle	1454 ± 379	711–2197	1730 ± 375	995–2465	< 0.001
Apical	1628 ± 430	785–2471	1794 ± 452	908–2680	< 0.001
Anterior WI (mmHg%)					
Basal	1575 ± 376	838–2312	1681 ± 414	870–2492	0.007
Middle	1461 ± 355	765–2157	1606 ± 377	867–2345	< 0.001
Apical	1473 ± 370	748–2198	1623 ± 389	861–2385	< 0.001
Average WI of the anteroseptal wall (mmHg%)	1650 ± 242	1176–2124	1722 ± 253	1226–2218	0.004
Average WI of the septal wall (mmHg%)	1734 ± 271	1203–2265	1876 ± 276	1335–2417	< 0.001
Average WI of the inferior wall (mmHg%)	1838 ± 311	1228–2448	1944 ± 323	1311–2577	0.001
Average WI of the posterior wall (mmHg%)	1726 ± 268	1201–2251	1873 ± 286	1312–2434	< 0.001
Average WI of the lateral wall (mmHg%)	1607 ± 299	1021–2193	1834 ± 307	1232–2436	< 0.001
Average WI of the anterior wall (mmHg%)	1503 ± 269	976–2030	1636 ± 294	1060–2212	< 0.001
Average WI of the basal level (mmHg%)	1628 ± 237	1163–2093	1779 ± 249	1291–2267	< 0.001
Average WI of the middle level (mmHg%)	1596 ± 246	1114–2078	1766 ± 253	1270–2262	< 0.001
Average WI of the apical level (mmHg%)	1804 ± 267	1281–2327	1897 ± 290	1329–2465	0.001

*CI* confidence interval, *SD* standard deviation; WI, work index. *P-value refers to sex differences

**Table 5:** Parameters of left ventricular segmental WE according to sex

	Men, median (IQR)	Men, limits of normality ± SE	Women, median (IQR)	Women, limits of normality ± SE	*P*-value*
Anteroseptal WE (%)					
Basal	91 (87–95)	75 ± 1.8	93 (89–96)	79 ± 1.8	0.004
Middle	97 (94–99)	84 ± 1.2	98 (96–99)	89 ± 1.1	0.026
Apical	98 (95–99)	84 ± 1.6	98 (96–99)	86 ± 1.5	0.619
Septal WE (%)					
Basal	95 (91–98)	78 ± 1.8	95 (92–98)	82 ± 1.2	0.582
Middle	95 (92–98)	82 ± 1.1	97 (95–98)	88 ± 1.2	< 0.001
Apical	98 (97–99)	90 ± 1.0	98 (97–99)	89 ± 1.6	0.858
Inferior WE (%)					
Basal	96 (94–98)	84 ± 1.0	96 (94–98)	83 ± 1.4	0.297
Middle	96 (93–98)	82 ± 1.4	97 (94–98)	86 ± 1.2	0.010
Apical	98 (96–99)	83 ± 2.1	98 (95–99)	88 ± 0.9	0.566
Posterior WE (%)					
Basal	95 (91–97)	83 ± 1.0	96 (93–97)	82 ± 1.8	0.038
Middle	96 (92–98)	80 ± 1.4	97 (94–99)	87 ± 0.9	0.001
Apical	97 (94–99)	86 ± 0.8	98 (94–99)	86 ± 1.3	0.403
Lateral WE (%)					
Basal	96 (94–98)	83 ± 1.8	96 (94–98)	87 ± 0.6	0.540
Middle	97 (93–98)	83 ± 1.3	98 (96–99)	87 ± 1.3	0.001
Apical	98 (95–99)	81 ± 1.8	98 (96–99)	86 ± 1.5	0.429
Anterior WE (%)					
Basal	96 (92–98)	84 ± 1.0	96 (92–98)	81 ± 1.8	0.305
Middle	97 (93–99)	83 ± 1.1	97 (95–99)	85 ± 1.1	0.369
Apical	97 (93–99)	79 ± 2.6	98 (95–99)	85 ± 1.3	0.024
Average WE of the anteroseptal wall (%)	95 (93–96)	85 ± 1.3	96 (94–97)	89 ± 0.7	0.001
Average WE of the septal wall (%)	95 (94–97)	89 ± 0.6	96 (94–98)	90 ± 0.6	0.001
Average WE of the inferior wall (%)	96 (94–97)	89 ± 0.6	96 (95–97)	90 ± 0.7	0.157
Average WE of the posterior wall (%)	95 (93–97)	88 ± 0.7	96 (95–97)	89 ± 0.7	< 0.001
Average WE of the lateral wall (%)	96 (94–98)	86 ± 1.0	97 (95–98)	91 ± 0.6	0.036
Average WE of the anterior wall (%)	96 (93–97)	85 ± 1.0	96 (94–98)	89 ± 0.4	0.087
Average WE of the basal level (%)	94 (92–95)	90 ± 0.4	95 (93–96)	89 ± 0.6	0.002
Average WE of the middle level (%)	96 (93–97)	86 ± 1.0	97 (95–98)	91 ± 0.7	< 0.001
Average WE of the apical level (%)	97 (96–98)	89 ± 0.9	97 (96–98)	91 ± 0.7	0.038

*IQR* interquartile range, *SE* standard error; WE, work efficiency. *P-value refers to sex differences

Except for the anteroseptal apical WI, inferior apical WI, and posterior apical WI, all WIs of different segments were lower in men than in women. Similarly, the average WI was significantly lower in men than in women between different levels as well as different walls.

WE was significantly different between sexes in some LV segments. Except for the average values of the inferior and anterior walls of WE, all average values of walls and levels of WE were higher in men than in women.

### Sex and age differences

Table 6, Supplement Fig. 1, and Supplement Fig. 2 show the sex and age differences in WI. Except for the septal middle WI increasing with age (*R*^2^ = 0.03, *P* = 0.017) and the inferior basal WI decreasing with age (*R*^2^ = 0.05, *P* = 0.001), there was no significant correlation between age and WI of the eighteen segments or the average WI of the varying walls and levels in men. However, eight of the eighteen segments’ WI increased with age in women. Moreover, except for the average WI of the septal and posterior walls, which showed no correlation with age, all average WIs of different walls and levels increased with age in women. There was no correlation between age groups and average WI of the different walls or levels in men; nevertheless, most of the average WI of different walls and levels in women showed significant differences between age groups. In Supplement Fig. 1 and Supplement Fig. 2, the sex differences in the WI of some segments, levels, and walls in the different age subgroups are shown.

**Table 6:** Parameters of left ventricular 18-segment myocardial WI and blood pressure according to sex and age

	< 30 years (*n* = 116)		30–40 years (*n* = 144)		40–50 years (*n* = 94)		≥ 50 years (*n* = 57)		*p*-value		Men		Women	
	Men (*n* = 59), mean ± SD or median (IQR)	Women (*n* = 57), mean ± SD or median (IQR)	Men (*n* = 72), mean ± SD or median (IQR)	Women (*n* = 72), mean ± SD or median (IQR)	Men (*n* = 41), mean ± SD or median (IQR)	Women (*n* = 53), mean ± SD or median (IQR)	Men (*n* = 23), mean ± SD or median (IQR)	Women (*n* = 34), mean ± SD or median (IQR)	Men	Women	*R*	*p*-value	*R*	*p*-value
SBP (mmHg)	120 (115–130)	115 (106–122)*	124 (114–130)	110 (100–117)*	126 (117–131)	115 (106–128)*	122 (117–132)	124 (112–131)	0.305	< 0.001	0.134	0.062	0.193	0.004
DSP (mmHg)	70 (63–74)	71 (63–75)	72 (66–77)	70 (62–76)	79 (73–84)	71 (63–80)*	75 (68–81)	74 (66–81)	< 0.001	0.138	0.354	< 0.001	0.118	0.083
Anteroseptal WI (mmHg%)														
Basal	1368 ± 344	1503 ± 334*	1365 ± 316	1464 ± 294^e^	1405 ± 314	1466 ± 330	1393 ± 220	1604 ± 351*	0.914	0.174	0.097	0.175	0.072	0.291
Middle	1653 ± 344	1787 ± 354* ^c^	1765 ± 326	1726 ± 354^d e^	1706 ± 358	1912 ± 287*	1749 ± 249	1977 ± 328*	0.262	0.001	0.084	0.243	0.196	0.004
Apical	1859 ± 406	1751 ± 438^b c^	1845 ± 387	1746 ± 342^d e^	1902 ± 404	1990 ± 356	1808 ± 392	1958 ± 354	0.815	< 0.001	0.014	0.843	0.245	< 0.001
Septal WI (mmHg%)														
Basal	1456 ± 284	1602 ± 337*	1459 ± 334	1531 ± 340^e^	1467 ± 354	1576 ± 285	1337 ± 359	1689 ± 409*	0.413	0.163	-0.047	0.514	0.039	0.564
Middle	1609 ± 299^c^	1831 ± 285* ^c^	1725 ± 350	1794 ± 306^e^	1702 ± 403	1876 ± 307* ^f^	1801 ± 280	2015 ± 354*	0.092	0.007	0.171	0.017	0.144	0.035
Apical	2056 ± 371	2178 ± 434	2022 ± 360	2148 ± 336*	2113 ± 333	2182 ± 375	2120 ± 377	2263 ± 423	0.506	0.563	0.040	0.577	0.068	0.318
Inferior WI (mmHg%)														
Basal	1804 ± 431^b c^	1847 ± 457^c^	1772 ± 421^e^	1825 ± 431^e^	1632 ± 378	1892 ± 437*	1525 ± 423	2052 ± 392*	0.017	0.080	-0.227	0.001	0.111	0.104
Middle	1713 (1389–2041)	1716 (1405–1940)^c^	1826 (1496–2086)	1822 (1534–2026)	1702 (1440–1999)	1860 (1774–2066)*	1740 (1595–2006)	2007 (1669–2322)*	0.498	0.003	0.051	0.478	0.247	< 0.001
Apical	2079 (1767–2388)	2120 (1734–2280)	2094 (1805–2329)	2112 (1829–2462)	2044 (1734–2423)	2076 (1889–2436)	2059 (1820–2307)	2164 (1852–2442)	0.970	0.784	-0.011	0.878	0.077	0.261
Posterior WI (mmHg%)														
Basal	1891 (1696–2189)	2053 (1815–2359)	1885 (1596–2174)	2055 (1803–2316)*	1997 (1625–2254)	2095 (1855–2319)	1698 (1565–2109)	2155 (1709–2313)*	0.324	0.944	-0.084	0.244	0.029	0.673
Middle	1495 (1282–1666)	1658 (1512–1967)*	1548 (1283–1786)	1773 (1490–1893)*	1425 (1230–1822)	1736 (1447–1955)*	1372 (1148–1870)	1916 (1577–2098)*	0.867	0.131	-0.034	0.634	0.070	0.309
Apical	1805 ± 368	1895 ± 396^a^	1718 ± 370	1738 ± 420	1796 ± 429	1874 ± 429	1687 ± 390	1851 ± 486	0.418	0.155	-0.052	0.473	-0.010	0.889
Lateral WI (mmHg%)														
Basal	1706 ± 353^c^	1875 ± 400* ^b c^	1720 ± 402	1884 ± 383* ^d e^	1736 ± 358	2093 ± 354*	1884 ± 267	2175 ± 312*	0.231	< 0.001	0.132	0.065	0.299	< 0.001
Middle	1470 (1149–1745)	1730 (1466–1903)*	1421 (1122–1668)	1631 (1443–1923)*	1420 (1269–1700)	1815 (1549–2108)*	1493 (1241–1606)	1788 (1696–2099)*	0.814	0.024	0.054	0.451	0.174	0.011
Apical	1589 (1327–1928)	1743 (1451–2133)	1480 (1314–1767)	1864 (1517–2072)*	1746 (1486–1989)	1756 (1432–2113)	1745 (1422–2128)	1882 (1538–2257)	0.067	0.689	0.112	0.118	0.051	0.459
Anterior WI (mmHg%)														
Basal	1593 (1385–1793)	1757 (1302–1853)	1594 (1353–1817)	1606 (1326–1938)	1529 (1240–1954)	1755 (1366–1953)	1620 (1260–2076)	1840 (1481–2189)	0.957	0.136	0.008	0.915	0.141	0.038
Middle	1479 ± 383	1502 ± 370^b c^	1433 ± 349	1560 ± 375* ^d e^	1490 ± 319	1695 ± 370*	1448 ± 376	1739 ± 350*	0.829	0.005	0.054	0.456	0.247	< 0.001
Apical	1416 (1235–1636)	1482 (1146–1876)^c^	1435 (1156–1736)	1562 (1338–1851)*	1538 (1299–1876)	1641 (1449–1974)	1474 (1162–1677)	1851 (1472–2059)*	0.504	0.012	0.085	0.236	0.231	0.001
Average WI of the anteroseptal wall (mmHg%)	1626 ± 231	1680 ± 260^b c^	1658 ± 248	1645 ± 237^d e^	1671 ± 265	1789 ± 226*	1650 ± 220	1846 ± 250*	0.814	< 0.001	0.084	0.241	0.243	< 0.001
Average WI of the septal wall (mmHg%)	1707 ± 257	1870 ± 272* ^c^	1735 ± 374	1824 ± 266^e^	1761 ± 296	1878 ± 267*	1753 ± 258	1989 ± 295*	0.781	0.040	0.081	0.262	0.126	0.065
Average WI of the inferior wall (mmHg%)	1850 ± 329	1885 ± 338^c^	1869 ± 309	1911 ± 325^e^	1804 ± 303	1973 ± 294*	1766 ± 288	2067 ± 313*	0.474	0.045	-0.079	0.270	0.188	0.006
Average WI of the posterior wall (mmHg%)	1789 (1565–1951)	1881 (1688–2073)*	1700 (1515–1879)	1899 (1644–2167)*	1721 (1486–1984)	1968 (1686–2059)*	1679 (1345–1777)	1962 (1629–2192)*	0.300	0.297	-0.083	0.249	0.041	0.549
Average WI of the lateral wall (mmHg%)	1616 (1421–1781)	1788 (1611–2002)* ^c^	1503 (1382–1791)	1822 (1615–2028)*	1638 (1470–1830)	1896 (1673–2049)*	1739 (1508–1847)	1939 (1764–2140)*	0.136	0.021	0.118	0.100	0.214	0.002
Average WI of the anterior wall (mmHg%)	1511 (1352–1616)	1548 (1349–1712)^c^	1481 (1269–1679)	1605 (1397–1830)*	1537 (1328–1737)	1665 (1510–1862)*	1471 (1305–1720)	1785 (1498–1992)*	0.746	0.003	0.061	0.398	0.266	< 0.001
Average WI of the basal level (mmHg%)	1646 ± 220	1752 ± 264* ^c^	1626 ± 248	1728 ± 251* ^e^	1626 ± 252	1800 ± 207*	1592 ± 226	1901 ± 243*	0.833	0.006	-0.066	0.360	0.181	0.008
Average WI of the middle level (mmHg%)	1568 ± 244	1702 ± 234* ^b c^	1610 ± 258	1711 ± 263* ^d e^	1598 ± 244	1814 ± 225*	1624 ± 226	1916 ± 231*	0.730	< 0.001	0.084	0.241	0.281	< 0.001
Average WI of the apical level (mmHg%)	1850 ± 359	1894 ± 373	1772 ± 262	1851 ± 280^e^	1859 ± 249	1941 ± 270	1797 ± 277	1971 ± 275*	0.423	0.127	0.075	0.299	0.157	0.021

*IQR* interquartile range, *SD* standard deviation, *WI* work index. *P-value < 0.05 vs. men. ^a^Significant difference between < 30 years of age and 30 to 40 years of age. ^b^Significant difference between < 30 years of age and 40 to 50 years of age. ^c^Significant difference between < 30 years of age and ≥ 50 years of age. ^d^Significant difference between 30 to 40 years of age and 40 to 50 years of age. ^e^Significant difference between 30 to 40 years of age and ≥ 50 years of age. ^f^Significant difference between 40 to 50 years of age and ≥ 50 years of age

Table 7, Supplement Fig. 3, and Supplement Fig. 4 show the sex and age differences in WE. Except for the posterior middle WE and posterior apical WE decreasing with age (*R*^2^ = 0.02, *P* = 0.036 and *R*^2^ = 0.02, *P* = 0.034, respectively) in men and the posterior basal WE increasing with age (*R*^2^ = 0.05, *P* = 0.001) and the posterior apical WE decreasing with age (*R*^2^ = 0.07, *P* < 0.001) in women, there was no correlation between age and WE of the different segments or the average WE of the varying walls and levels in either men or women. There was no correlation between age groups and average WE of the varying walls and levels in either men or women. In Supplement Fig. 3 and Supplement Fig. 4, WE for only a few of the different segments, levels, and walls in the different age subgroups showed sex differences.

**Table 7:** Parameters of left ventricular 18-segment myocardial WE according to sex and age

	< 30 years (n = 116)		30–40 years (n = 144)		40–50 years (*n* = 94)		≥ 50 years (*n* = 57)		*p*-value		Men		Women	
	Men (n = 59), median (IQR)	Women (n = 57), median (IQR)	Men (n = 72), median (IQR)	Women (n = 72), median (IQR)	Men (n = 41), median (IQR)	Women (n = 53), median (IQR)	Men (n = 23), median (IQR)	Women (n = 34), median (IQR)	Men	Women	*R*	*p*-value	*R*	*p*-value
Anteroseptal WE (%)														
Basal	93 (88–94)	94 (89–98)	91 (87–95)	93 (89–95)	91 (86–96)	94 (90–97)*	89 (87–93)	91 (86–96)	0.496	0.139	-0.118	0.101	-0.102	0.136
Middle	97 (94–98)	98 (96–99)*	97 (95–99)	98 (95–99)	98 (94–99)	98 (96–99)	97 (94–99)	97 (95–99)	0.851	0.738	0.013	0.857	-0.041	0.550
Apical	98 (95–99)	97 (96–99)	97 (96–99)	98 (96–99)	98 (96–99)	97 (96–99)	98 (95–99)	98 (96–99)	0.755	0.314	0.016	0.829	0.028	0.687
Septal WE (%)														
Basal	95 (91–97)	96 (92–98)	96 (93–98)^c^	96 (92–98)	95 (90–97)	95 (92–97)	92 (87–95)	95 (91–97)	0.046	0.350	-0.083	0.250	-0.129	0.059
Middle	95 (90–97)	97 (94–98)*	95 (92–98)	98 (95–99)*	96 (91–98)	97 (94–98)	96 (93–97)	97 (95–99)	0.580	0.230	0.094	0.193	-0.020	0.773
Apical	98 (97–99)	98 (96–99)	98 (95–99)	98 (97–99)	99 (97–99)	98 (98–99)	99 (98–99)	98 (97–99)	0.051	0.978	0.109	0.131	-0.027	0.694
Inferior WE (%)														
Basal	96 (94–97)	97 (94–99)	96 (94–98)	97 (94–98)	96 (93–98)	96 (93–98)	95 (92–98)	96 (93–98)	0.689	0.314	-0.016	0.821	-0.122	0.074
Middle	96 (93–98)	96 (92–98)	95 (92–98)	96 (94–98)*	96 (93–98)	97 (95–98)	95 (92–98)	98 (95–98)	0.739	0.704	-0.010	0.890	0.052	0.447
Apical	98 (96–99)	98 (95–99)	98 (95–99)	98 (95–99)	98 (96–99)	98 (96–99)	98 (94–98)	97 (95–98)	0.341	0.281	-0.030	0.676	-0.080	0.239
Posterior WE (%)														
Basal	94 (91–97)	94 (92–97)	95 (91–97)	95 (93–98)	96 (92–98)	96 (94–98)	93 (90–97)	96 (94–98)	0.386	0.056	0.063	0.381	0.219	0.001
Middle	97 (93–99)	97 (93–98)	96 (93–98)	98 (95–99)*	95 (90–98)	97 (94–99)	93 (86–97)	98 (96–99)*	0.096	0.190	-0.150	0.036	0.068	0.317
Apical	97 (95–99)	99 (96–99)* ^a b^	98 (95–99)	98 (95–99)^c^	96 (94–99)	97 (94–99)	95 (88–99)	95 (93–98)	0.081	< 0.001	-0.152	0.034	-0.271	< 0.001
Lateral WE (%)														
Basal	96 (93–98)	96 (93–98)	97 (94–98)	97 (94–98)	97 (94–98)	96 (95–99)	96 (93–99)	96 (95–97)	0.775	0.881	0.071	0.324	0.065	0.343
Middle	97 (93–99)	97 (94–99)	97 (92–99)	98 (95–99)*	97 (95–98)	98 (96–99)	97 (95–98)	98 (96–99)*	0.410	0.174	-0.093	0.194	0.104	0.127
Apical	98 (95–99)	97 (96–99)	97 (94–99)	98 (96–99)*	98 (96–99)	98 (96–99)	98 (97–99)	97 (93–99)	0.152	0.444	0.051	0.483	-0.036	0.600
Anterior WE (%)														
Basal	96 (93–98)	97 (92–99)	96 (92–97)	96 (93–98)	95 (91–98)	96 (94–98)	96 (93–97)	94 (92–97)	0.299	0.123	-0.101	0.160	-0.086	0.209
Middle	97 (93–99)	96 (94–99)	97 (90–99)	98 (95–99)	97 (94–99)	97 (95–99)	96 (93–98)	96 (95–98)	0.832	0.358	-0.039	0.588	-0.007	0.913
Apical	96 (92–98)	96 (94–99)	96 (92–99)	98 (95–99)*	98 (95–99)	98 (94–99)	96 (92–98)	98 (95–99)	0.024	0.192	0.083	0.250	0.085	0.212
Average WE of the anteroseptal wall (%)	95 (93–97)	96 (94–97)	95 (92–96)	96 (93–97)*	95 (92–97)	96 (94–97)	95 (93–96)	95 (92–97)	0.873	0.303	-0.060	0.402	-0.050	0.466
Average WE of the septal wall (%)	96 (94–97)	97 (94–98)*	96 (93–97)	96 (95–98)*	95 (94–97)	96 (95–97)	95 (94–96)	96 (94–98)	0.885	0.618	0.027	0.713	-0.089	0.194
Average WE of the inferior wall (%)	96 (94–97)	96 (94–98)	96 (94–97)	97 (95–98)	96 (95–98)	96 (95–98)	95 (93–97)	96 (95–97)	0.524	0.573	-0.023	0.749	-0.018	0.795
Average WE of the posterior wall (%)	95 (93–97)	96 (95–97)	95 (94–97)	96 (95–97)*	95 (93–97)	96 (95–97)	94 (90–96)	96 (95–97)*	0.084	0.577	-0.122	0.089	0.018	0.787
Average WE of the lateral wall (%)	97 (93–98)	96 (94–97)	96 (93–97)	97 (95–98)*	97 (95–98)	97 (96–98)	96 (95–97)	97 (95–98)	0.337	0.345	0.032	0.661	0.089	0.190
Average WE of the anterior wall (%)	96 (94–97)	96 (93–98)	96 (93–97)	97 (94–98)*	96 (93–97)	96 (94–98)	95 (93–97)	96 (93–97)	0.615	0.151	-0.042	0.564	0.009	0.900
Average WE of the basal level (%)	94 (92–96)	95 (93–96)	95 (93–95)	95 (94–96)	94 (92–96)	95 (93–96)	93 (92–95)	94 (93–96)	0.342	0.509	-0.071	0.326	-0.010	0.881
Average WE of the middle level (%)	96 (93–97)	96 (94–98)	96 (94–97)	97 (96–98)*	95 (94–98)	97 (95–98)*	95 (93–96)	97 (95–98)*	0.745	0.154	-0.064	0.374	0.103	0.133
Average WE of the apical level (%)	97 (96–98)	97 (96–98)	96 (95–98)	98 (96–98)*	97 (96–98)	97 (96–98)	97 (95–98)	97 (95–98)	0.172	0.139	0.063	0.379	-0.058	0.395

*IQR* interquartile range, *WE* work efficiency. *P-value < 0.05 vs. men. ^a^Significant difference between < 30 years of age and 40 to 50 years of age. ^b^Significant difference between < 30 years of age and ≥ 50 years of age. ^c^Significant difference between 30 to 40 years of age and ≥ 50 years of age

### Repeatability and reproducibility

Intra- and inter-observer variabilities for WI and WE of the eighteen segments are summarized in Supplement Table 1, Supplement Fig. 5, Supplement Fig. 6, Supplement Fig. 7, and Supplement Fig. 8. Good intra-observer and inter-observer reproducibility were found.

## Discussion

This study is the first to use echocardiography to analyse 18-segment myocardial WI and WE of noninvasive LVMW. The LVMW, derived from the LVPSL, was first derived by Russell et al. [1] as a novel method to assess LV function. The study showed that LV myocardial glucose metabolism (calculated by positron emission tomography) has a strong correlation with noninvasive LVMW. Recently, Edwards et al. [11] revealed that in patients with normal wall motion and ejection fraction, noninvasive LVMW was more sensitive than global LS to detect significant coronary artery disease. These studies revealed that afterload-enrolled noninvasive LVMW could be a reliable method to evaluate LV function.

Multiple studies have already concluded normal LVMW by echocardiography; nevertheless, they only evaluated global myocardial work [14–16]. In our study, the LV GLS was higher in women than in men, which parallels the results from other studies [8, 17]. The LV global WI and LV global CW were higher in women than in men; a possible reason could be that the LV global WI and the LV global CW are correlated with the LV GLS [18]. The LV global WI was significantly lower in the present study than it was in a previous study [16], racial differences being a possible reason for the discrepancy (1749 mmHg ± 231 vs. 1896 mmHg ± 308, *P* < 0.001), though the LV global WE was similar in the two studies.

Our study establishes normal reference values for LV 18-segment WI and WE in a healthy Asian population. The data demonstrated that there are differences in WI and WE between different segments, sexes and age groups. The study further strengthens the necessity for the segment-, sex-, and age-specific normal ranges of WI and WE.

### Functional nonuniformity

Functional heterogeneity, as a well-known feature of the left ventricle in the normal population, may influence LV segmental function [19–22]. In this study, as we expected, an important observation in evaluating the entire population was the variability of WI and WE for different segments, levels, and walls of the left ventricle.

Notably, the anteroseptal basal segment had the lowest WI and WE among all segments. Based on our data, the basal and middle levels demonstrated lower median WI values than the apical level in all walls. The reason could be that WI is significantly correlated with LV GLS, and the strain of apical levels is greater than the strain of middle and basal levels [23]. WI was lower in anterior walls than in the other walls at all levels, which may be the result of the strain of anterior walls being lower than that of the other walls in the normal population [24]. WE showed a lower median value in the basal wall than in the other walls at all levels. Moreover, all 18-segment median values of WI were greater than 95%, except for the anteroseptal basal segment.

### Sex and age differences

Our data showed that most WI values were independent of sex (Table 4). In the segments with significant differences, the WI values of males were higher than those of females. The average WI values of different levels and walls were all higher in men than in women. This may be related to the result that LV GLS is higher in women than in men. Moreover, when considering sex and age, all the average WIs of different walls and levels showed no correlation with age in men (Table 6). However, most of the average WI of different walls and levels increased with age in women along with systolic blood pressure. The results above are consistent with the study by Manganaro et al. [16], who demonstrated that increasing afterload may lead to higher WI. Therefore, the ageing-related increase in systolic blood pressure may be the reason for the increase in WI in some segments in women.

There were some differences in WE between the sexes (Table 5). The average WE of all levels and most walls were significantly different in men and women. When both sex and age were considered, none of the average WE values of the different walls or levels showed any correlation with age in women or men (Table 7).

### Clinical implications

To our knowledge, LVMW has been studied in the fields of heart failure, hypertension, cardiac resynchronization therapy, diabetes mellitus, cardiomyopathy (nonobstructive hypertrophic cardiomyopathy [HCM], dilated cardiomyopathy [DCM], cardiac amyloidosis [CA]), etc. [2–7]. As a noninvasive and novel technique, LVMW could be a reliable method to measure different LV segmental functions in clinical and experimental research.

Coronary artery disease (CAD) is a leading disease worldwide [25]. Boe et al. [26] previously demonstrated that the presence of ≥ 4 adjacent segments with systolic dysfunction (based on WI measurements) showed better sensitivity and specificity in identifying non-ST-segment elevation-acute coronary syndrome than conventional echocardiography parameters. In another study [11], Edwards et al. found that relative segmental WI decreased in the presence of segmental perfusion defects.

HCM is an inherited cardiovascular disease characterized by the presence of thick LV walls [27]. Hiemstra et al. [4] evaluated segmental differences in myocardial work in patients with nonobstructive HCM, and WE for some segments was significantly lower in patients with nonobstructive HCM than in control subjects.

DCM is a common cardiac disease with LV systolic dysfunction caused by many factors [28]. Recently, Schrub et al. [29] analysed the relationship between WE and exercise tolerance in patients with DCM. They demonstrated that septal WE was the best predictor of exercise performance in patients with DCM.

CA has a high incidence rate in elderly individuals [30, 31]. Clemmensen et al. [32] demonstrated that WI in apical, middle, and basal myocardial levels were all lower in patients with CA than in controls. WI gradually decreased from the apical level to the basal level in patients with CA. Moreover, another study [5] by Clemmensen et al. demonstrated that the apical-to-basal WI ratio could predict major adverse cardiac events and all-cause mortality in patients with CA.

Overall, our data showed good agreement and reproducibility in assessing WI and WE of different segments, which suggests the possibility that these normal values could be used as a reference for a variety of diseases in clinical and research practice, such as myocardial infarction, nonobstructive HCM, DCM, and CA.

### Limitations

The collection and quantification of the LVMW dataset from a single-provider platform may limit the application of the reference values to data measured by other provider platforms. In addition, the software only provides the values of WI and WE of each segment but does not provide the values of CW and WW of each segment, so the reference values for each segment of CW and WW are not available. Additionally, all enrolled individuals were asymptomatic on routine examinations, but the possibility of subclinical cardiovascular diseases, especially in elderly individuals, cannot be ruled out. Furthermore, whether our results apply to non-Asian populations remains unknown.

## Conclusions

To date, this study is the first to use echocardiography to establish reference values for the segment-, sex-, and age-specific normal ranges of WI and WE in a large normal population cohort. There are differences in WI and WE between different segments, levels, and walls of the normal left ventricle. Sex should be considered when attempting to identify WI and WE. Age should be considered when attempting to identify WI in women. The data in this study could enhance the value of echocardiography in LV function evaluation, disease diagnosis risk stratification, and prognosis.

## Supplementary Information

**Additional file 1: Supplement Figure 1.** Individual values of left ventricular 18-segment myocardial work index according to sex and age categories. Horizontal lines represent median values or mean values, appropriately. *P value < 0.05 between sexes. ^#^*P *value < 0.05 between age subgroups.

**Additional file 2: Supplement Figure 2.** Individual values of the left ventricular 6-wall and 3-level average myocardial work index according to sex and age categories. Horizontal lines represent median values or mean values, appropriately. *P value < 0.05 between sexes. ^#^*P *value < 0.05 between age subgroups.

**Additional file 3: Supplement Figure 3.** Individual values of left ventricular 18-segment myocardial work efficiency according to sex and age categories. Horizontal lines represent median values. *P value < 0.05 between sexes. ^#^*P *value < 0.05 between age subgroups.

**Additional file 4: Supplement Figure 4.** Individual values of left ventricular 6-wall and 3-level average myocardial work efficiency according to sex and age categories. Horizontal lines represent median values. *P value < 0.05 between sexes. ^#^*P *value < 0.05 between age subgroups.

**Additional file 5: Supplement Figure 5.** The Bland–Altman analysis for assessing intra-observer variability of myocardial work index of eighteen segments.

**Additional file 6: Supplement Figure 6.** The Bland–Altman analysis for assessing intra-observer variability of myocardial work efficiency of eighteen segments.

**Additional file 7: Supplement Figure 7.** The Bland–Altman analysis for assessing inter-observer variability of myocardial work index of eighteen segments.

**Additional file 8: Supplement Figure 8.** The Bland–Altman analysis for assessing inter-observer variability of myocardial work efficiency of eighteen segments.

**Additional file 9: Supplement Table 1.** Intra- and inter-observer variabilities of myocardial WI and WE.

## Data Availability

The data and material underlying this article will be shared on reasonable request to the corresponding authors.

## Abbreviations

LV: Left ventricular
WI: Work index
WE: Work efficiency
LVPSL: LV pressure-strain loop
LVMW: LV myocardial work
LS: Longitudinal strain
2DE: Two-dimensional echocardiography
4DE: Four-dimensional echocardiography
CW: Constructive work
IVR: Isovolumic relaxation
WW: Wasted work
SD: Standard deviation
LV GLS: LV global LS
HCM: Hypertrophic cardiomyopathy
DCM: Dilated cardiomyopathy
CA: Cardiac amyloidosis
CAD: Coronary artery disease
ACO: Acute coronary artery occlusion
