# Supplementary figures and images for: Echocardiographic reference ranges for noninvasive left ventricular 18-segment myocardial work index and work efficiency in a healthy Asian population
Source: Cardiovasc Ultrasound. 2023 Jan 23;21:2. doi: 10.1186/s12947-023-00299-4 (PMC9869544; doi:10.1186/s12947-023-00299-4)

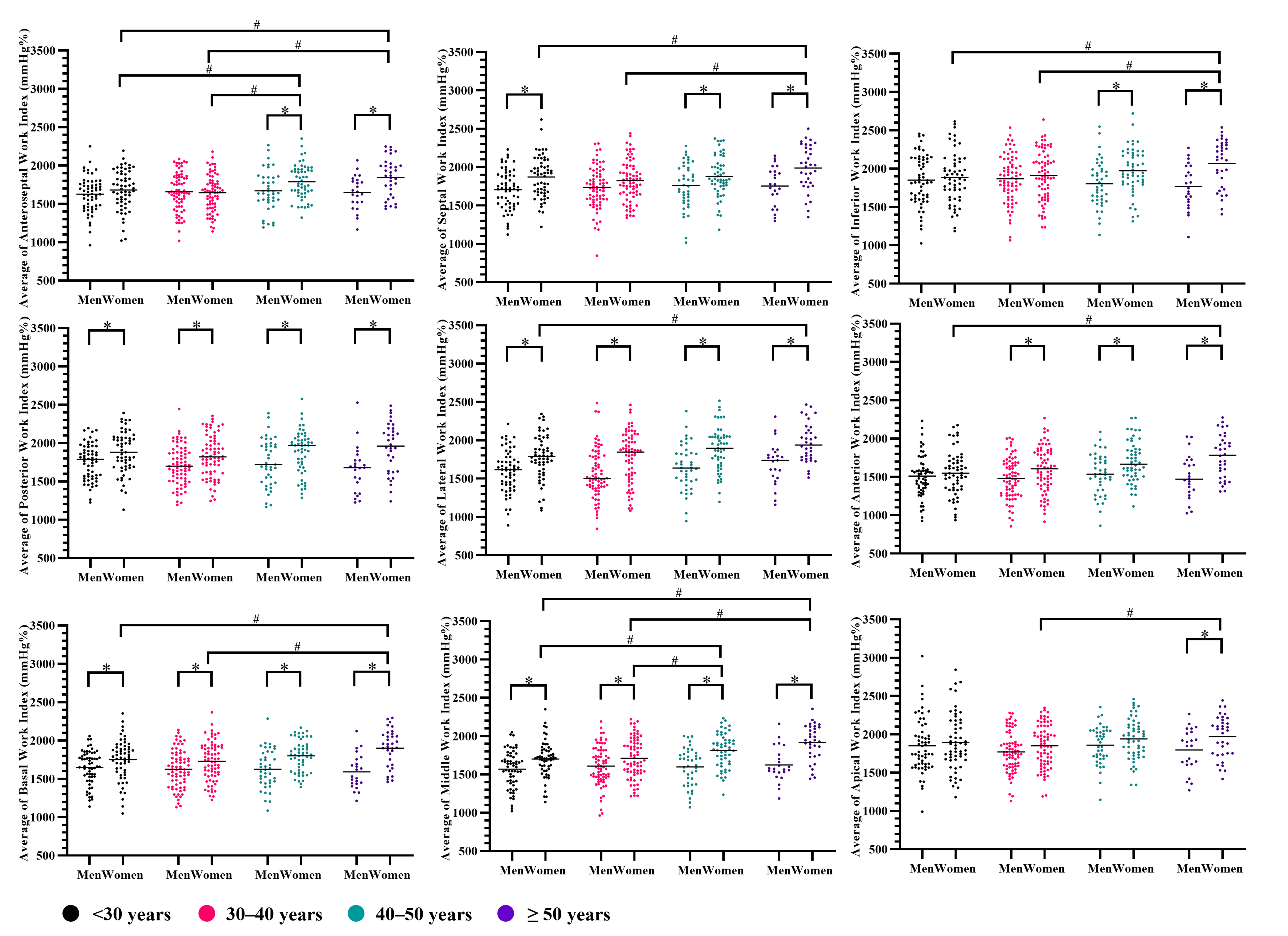

Supplement: Supplementary file 2 — Additional file 2: Supplement Figure 2. Individual values of the left ventricular 6-wall and 3-level average myocardial work index according to sex and age categories. Horizontal lines represent median values or mean values, appropriately. *P value < 0.05 between sexes. #P value < 0.05 between age subgroups. [file 12947_2023_299_MOESM2_ESM.tif]

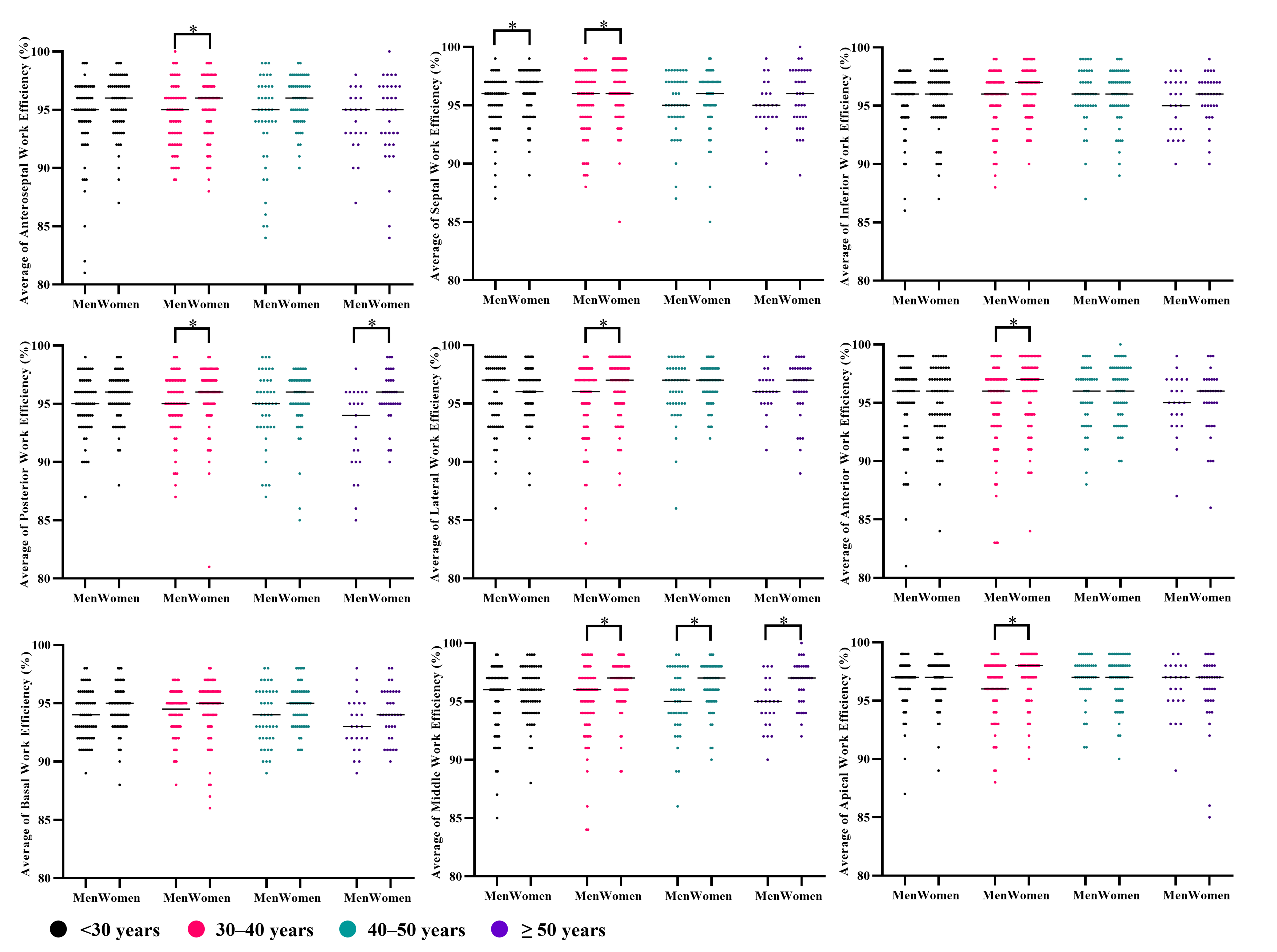

Supplement: Supplementary file 4 — Additional file 4: Supplement Figure 4. Individual values of left ventricular 6-wall and 3-level average myocardial work efficiency according to sex and age categories. Horizontal lines represent median values. *P value < 0.05 between sexes. #P value < 0.05 between age subgroups. [file 12947_2023_299_MOESM4_ESM.tif]

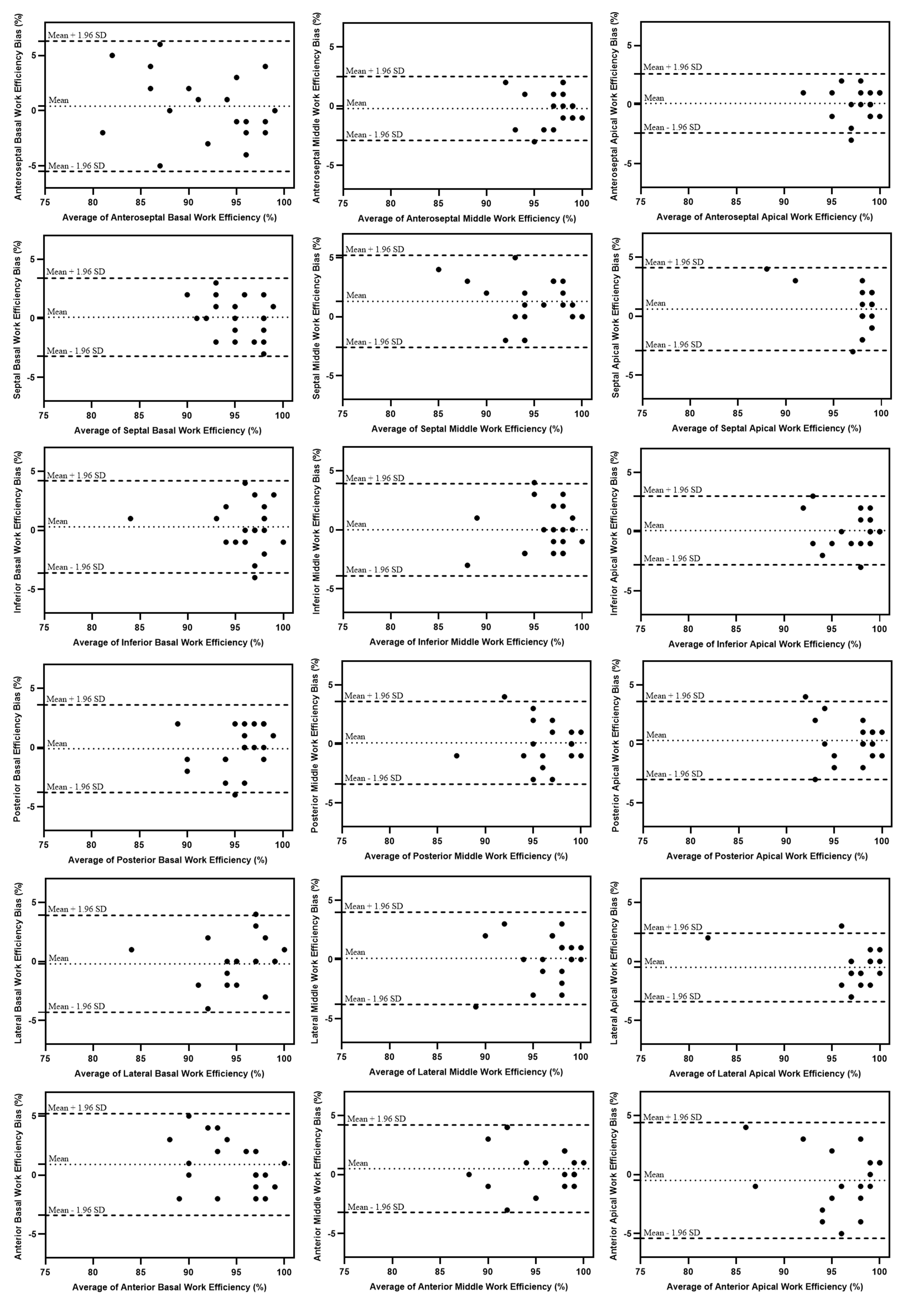

Supplement: Supplementary file 6 — Additional file 6: Supplement Figure 6. The Bland–Altman analysis for assessing intra-observer variability of myocardial work efficiency of eighteen segments. [file 12947_2023_299_MOESM6_ESM.tif]

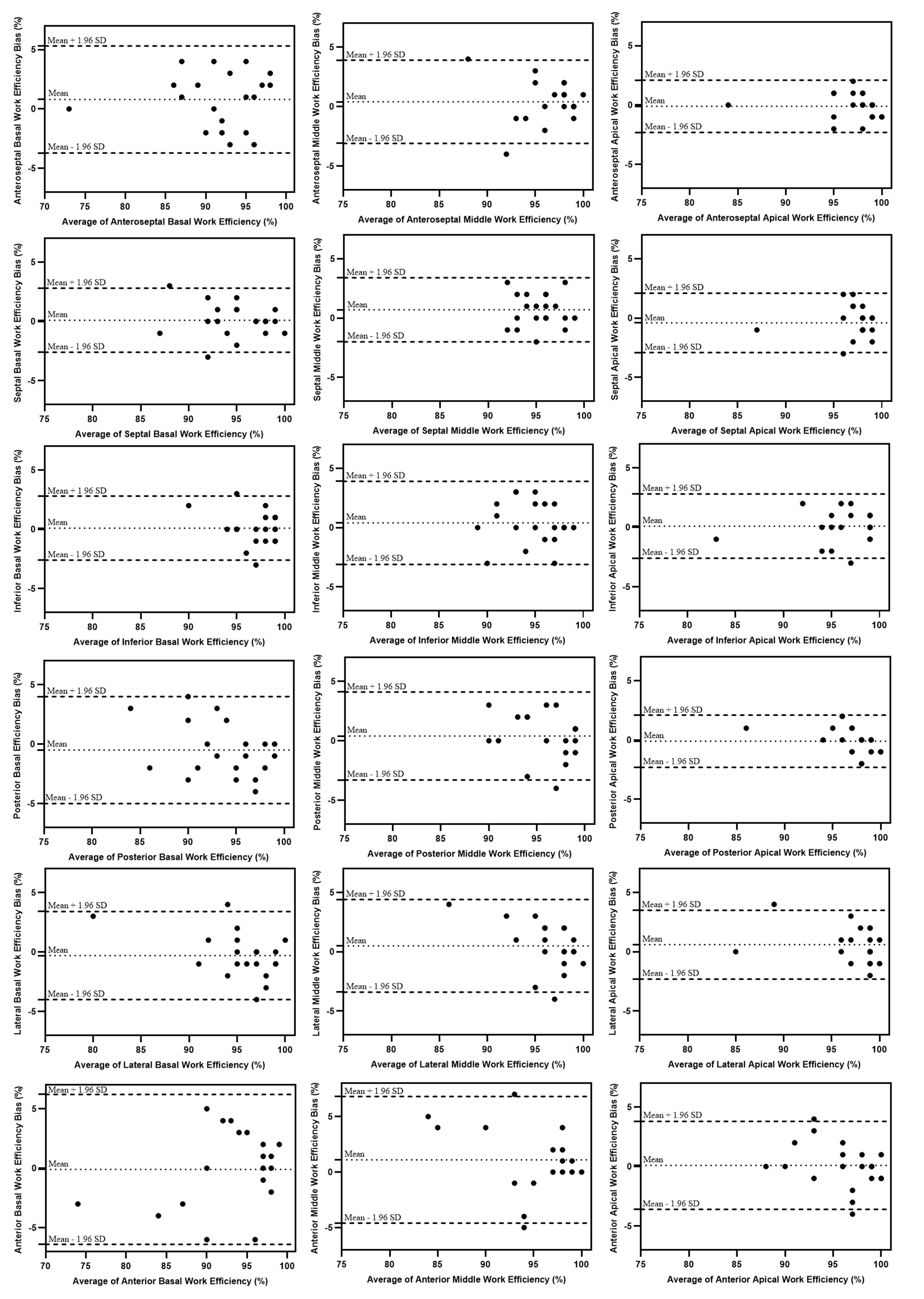

Supplement: Supplementary file 8 — Additional file 8: Supplement Figure 8. The Bland–Altman analysis for assessing inter-observer variability of myocardial work efficiency of eighteen segments. [file 12947_2023_299_MOESM8_ESM.tif]
