# Supplementary material for: Echocardiographic reference ranges for noninvasive left ventricular 18-segment myocardial work index and work efficiency in a healthy Asian population
Source: Cardiovasc Ultrasound. 2023 Jan 23;21:2. doi: 10.1186/s12947-023-00299-4 (PMC9869544; doi:10.1186/s12947-023-00299-4)
Supplement: Supplementary file 9 — Additional file 9: Supplement Table 1. Intra- and inter-observer variabilities of myocardial WI and WE. [file 12947_2023_299_MOESM9_ESM.docx]

| Supplement Table 1 Intra- and inter-observer variabilities of myocardial WI and WE | | | | | | |
| --- | --- | --- | --- | --- | --- | --- |
|  | Intra-observer variability | | | Inter-observer variability | | |
|  | Bias | 95% CI | ICC | Bias | 95% CI | ICC |
| Anteroseptal WI (mmHg%) |  |  |  |  |  |  |
| Basal | 22.1 | -210.4–254.6 | 0.95 | 80.2 | -134.4–294.8 | 0.94 |
| Middle | 26.6 | -136.7–189.9 | 0.96 | 34.9 | -193.0–262.8 | 0.94 |
| Apical | 27.3 | -175.0–229.6 | 0.98 | 32.8 | -159.9–225.5 | 0.96 |
| Septal WI (mmHg%) |  |  |  |  |  |  |
| Basal | -14.6 | -215.5–186.3 | 0.95 | 20.1 | -166.1–206.3 | 0.95 |
| Middle | 47.6 | -163.1–258.3 | 0.94 | 51.1 | -163.4–265.6 | 0.93 |
| Apical | 30.4 | -199.7–260.5 | 0.96 | -16.5 | -231.9–198.9 | 0.98 |
| Inferior WI (mmHg%) |  |  |  |  |  |  |
| Basal | 81.5 | -83.1–246.1 | 0.98 | 12.9 | -210.9–236.7 | 0.95 |
| Middle | 31.3 | -175.7–238.3 | 0.95 | 18.3 | -240.8–277.4 | 0.93 |
| Apical | 87.2 | -109.4–283.8 | 0.98 | 6.0 | -256.6–268.6 | 0.98 |
| Posterior WI (mmHg%) |  |  |  |  |  |  |
| Basal | 35.5 | -182.8–253.8 | 0.94 | -22.7 | -286.5–241.1 | 0.94 |
| Middle | 9.4 | -199.7–218.5 | 0.94 | 21.2 | -224.2–266.6 | 0.90 |
| Apical | 87.0 | -83.5–257.5 | 0.99 | 3.4 | -249.4–256.2 | 0.96 |
| Lateral WI (mmHg%) |  |  |  |  |  |  |
| Basal | 14.6 | -237.7–266.9 | 0.93 | -11.3 | -278.6–256.0 | 0.93 |
| Middle | 36.9 | -161.5–235.3 | 0.97 | 7.3 | -264.6–279.2 | 0.92 |
| Apical | 13.1 | -200.0–226.2 | 0.97 | 42.6 | -159.1–244.3 | 0.97 |
| Anterior WI (mmHg%) |  |  |  |  |  |  |
| Basal | -13.9 | -230.7–202.9 | 0.93 | -15.9 | -302.6–270.8 | 0.94 |
| Middle | 52.9 | -94.9–200.7 | 0.96 | 38.7 | -236.9–314.3 | 0.95 |
| Apical | 37.7 | -168.1–243.5 | 0.97 | 18.1 | -235.7–271.9 | 0.97 |
| Anteroseptal WE (%) |  |  |  |  |  |  |
| Basal | 0.4 | -5.5–6.3 | 0.86 | 0.8 | -3.7–5.3 | 0.92 |
| Middle | -0.2 | -2.9–2.5 | 0.81 | 0.4 | -3.1–3.9 | 0.84 |
| Apical | 0.1 | -2.4–2.6 | 0.83 | -0.1 | -2.3–2.1 | 0.95 |
| Septal WE (%) |  |  |  |  |  |  |
| Basal | 0.1 | -3.2–3.4 | 0.82 | 0.1 | -2.6–2.8 | 0.93 |
| Middle | 1.3 | -2.6–5.2 | 0.88 | 0.7 | -2.0–3.4 | 0.81 |
| Apical | 0.6 | -2.9–4.1 | 0.81 | -0.4 | -2.9–2.1 | 0.88 |
| Inferior WE (%) |  |  |  |  |  |  |
| Basal | 0.3 | -3.6–4.2 | 0.84 | 0.1 | -2.6–2.8 | 0.82 |
| Middle | 0.0 | -3.9–3.9 | 0.82 | 0.4 | -3.1–3.9 | 0.82 |
| Apical | 0.1 | -2.8–3.0 | 0.84 | 0.1 | -2.6–2.8 | 0.93 |
| Posterior WE (%) |  |  |  |  |  |  |
| Basal | -0.1 | -3.8–3.6 | 0.80 | -0.5 | -5.0–4.0 | 0.86 |
| Middle | 0.1 | -3.4–3.6 | 0.85 | 0.4 | -3.3–4.1 | 0.82 |
| Apical | 0.3 | -3.0–3.6 | 0.80 | -0.1 | -2.3–2.1 | 0.93 |
| Lateral WE (%) |  |  |  |  |  |  |
| Basal | -0.2 | -4.3–3.9 | 0.85 | -0.3 | -4.0–3.4 | 0.91 |
| Middle | 0.1 | -3.8–4.0 | 0.81 | 0.5 | -3.4–4.4 | 0.82 |
| Apical | -0.5 | -3.4–2.4 | 0.92 | 0.6 | -2.3–3.5 | 0.92 |
| Anterior WE (%) |  |  |  |  |  |  |
| Basal | 0.9 | -3.4–5.2 | 0.84 | -0.1 | -6.4–6.2 | 0.87 |
| Middle | 0.5 | -3.2–4.2 | 0.88 | 1.1 | -4.6–6.8 | 0.82 |
| Apical | -0.5 | -5.4–4.4 | 0.82 | 0.1 | -3.6–3.8 | 0.86 |
| CI, confidence interval; ICC, intraclass correlation coefficient; WE, work efficiency; WI, work index. | | | | | | |
